# Isolation and characterization of dengue virus serotype 2 from the large dengue outbreak in Guangdong, China in 2014

**DOI:** 10.1007/s11427-014-4782-3

**Published:** 2014-12-11

**Authors:** Hui Zhao, LingZhai Zhao, Tao Jiang, XiaoFeng Li, Hang Fan, WenXin Hong, Yu Zhang, Qin Zhu, Qing Ye, YiGang Tong, WuChun Cao, FuChun Zhang, ChengFeng Qin

**Affiliations:** 1 State Key Laboratory of Pathogen and Biosecurity, Beijing Institute of Microbiology and Epidemiology, Beijing, 100071 China; 2 Guangzhou 8th People’s Hospital, Guangzhou Medical University, Guangzhou, 510060 China

**Keywords:** dengue virus serotype 2, virus isolation, phylogenetic analysis, envelope gene

## Abstract

Dengue has been well recognized as a global public health threat, but only sporadic epidemics and imported cases were reported in recent decades in China. Since July 2014, an unexpected large dengue outbreak has occurred in Guangdong province, China, resulting in more than 40000 patients including six deaths. To clarify and characterize the causative agent of this outbreak, the acute phase serum from a patient diagnosed with severe dengue was subjected to virus isolation and high-throughput sequencing (HTS). Traditional real-time RT-PCR and HTS with Ion Torrent PGM detected the presence of dengue virus serotype 2 (DENV-2). A clinical DENV-2 isolate GZ05/2014 was obtained by culturing the patient serum in mosquito C6/36 cells. The complete genome of GZ05/2014 was determined and deposited in GenBank under the access number KP012546. Phylogenetic analysis based on the complete envelope gene showed that the newly DENV-2 isolate belonged to Cosmopolitan genotype and clustered closely with other Guangdong strains isolated in the past decade. No amino acid mutations that are obviously known to increase virulence or replication were identified throughout the genome of GZ05/2014. The high homology of Guangdong DENV-2 strains indicated the possibility of establishment of local DENV-2 circulation in Guangdong, China. These results help clarify the origin of this epidemic and predict the future status of dengue in China.

**Electronic Supplementary Material:**

Supplementary material is available for this article at 10.1007/s11427-014-4782-3 and is accessible for authorized users.

Dengue has become the most widespread mosquito-borne virus disease, and the number and severity of dengue outbreaks have increased in recent three decades. Some 3.9 billion people living in tropical and sub-tropical regions are at risk of dengue virus (DENV) infection, resulting in 500000 severe dengue cases that require hospitalization each year [1]. DENV infection in humans can cause a wide range of clinical manifestations, from mild febrile disease to potentially fatal dengue shock syndrome [2]. DENV can be classified into four serotypes (DENV-1 to DENV-4), and the progression to more serious disease is frequently associated with secondary infection by heterologous serotypes [3]. No approved vaccine or specific drug is currently available for the prevention and treatment of dengue. Current efforts to curb dengue transmission depend entirely on control of the mosquito vectors or interruption of human-mosquito contact [4].

In mainland China, dengue fever is still characterized as an imported epidemic disease, and so far has not been con-

†Contributed equally to this work
*Corresponding author (email: qincf@bmi.ac.cn; gz8hzfc@126.com)

firmed to be endemic [5]. However, the threat of dengue epidemics in China is increasing, due to the rapid urbanization and environmental and climate factors [6]. Guangdong province lies in southeastern China, characterized with humid subtropical climate, where the *Aedes* mosquitos are widely distributed and can breed throughout the year. Guangdong is the major affected area by DENV in mainland China, and sporadic epidemics caused by imported cases have been frequently reported in the past decades [7].

Since July 2014, an unexpected large dengue epidemic has occurred in Guangdong, China. Till 31 October, a total of 44497 dengue fever cases have been reported, resulting in six deaths. It is urgently needed to isolate and characterize the circulating pathogen responsible for this epidemic. In the present study, we isolated a clinical DENV-2 strain from the acute phase serum of a patient with severe dengue, and the genetic characterization of this newly isolate has provided useful information to understand the origin of this epidemic.

## 1 Materials and methods

### 1.1 Case description and laboratory diagnosis

On 30 August 2014, an 87-year-old female patient, resident of Guangzhou city, with a history of encephalatrophy, renal calculus and cholecystolithiasis, was referred for admission in Guangzhou 8th People's Hospital, with complaints of fever, chill, severe myalgia and confusion. She suddenly fell ill three days before hospitalization. Acute and convalescent phase sera were collected for standard blood examination and molecular diagnosis. The patient was managed empirically by fluid therapy; all symptoms disappeared and laboratory parameters put back on 11 days post hospitalization. She finally recovered and was discharged on 12 September 2014.

The acute phase serum from the patient was subjected to real-time RT-PCR assay (DAAN, China) and IgM and IgG capture ELISA (PanBio, Australia) according to the manufacturers' instructions. The convalescent phase serum was collected for immune fluorescence assay to confirm the virus isolate. The study was approved by the Review Board of Guangzhou 8th People's Hospital, and informed consent was obtained from the patient.

### 1.2 Virus isolation and identification

Acute phase serum from the patient was inoculated in C6/36 mosquito cells and maintained in 1640 medium (Life Technologies, USA) supplemented with 2% fetal bovine serum (Life Technologies) at 28°C in 5% $CO_2$. When complete cytopathic effects (CPE) were observed [8,9], culture supernatant was collected and stored at −70°C until use. Indirect immunofluorescence assay (IFA) was performed with the convalescent phase serum as previously described [10]. In brief, confluent BHK-21 cells were infected with new isolate GZ05/2014. Cells were harvested 4 days post infection, and suspended in DMEM containing 10% FBS. The suspended cells were then inoculated onto slides and incubated for 6 h at 37°C to be adherent. Following this, the slides were rinsed and fixed with acetone for 30 min in −20°C. The patient's convalescent phase serum diluted (100 fold) in PBS was added on it. After incubation for 1 h at 37°C, the slides were washed three times in PBS. Then, Alexa Fluor®488 conjugated goat anti-mouse IgG (Life Technologies) was added and incubated for 30 min at 37°C. DAPI was used for nucleus staining for 5 min at room temperature. Finally, positive cells were detected using a fluorescence microscope (Olympus, Japan).

### 1.3 High-throughput sequencing (HTS)

Total RNAs from 400 μL virus culture supernatant were extracted with High Pure Viral RNA Kit (Roche, Germany), and mixed with 1 μL 10× RNase III reaction buffer and 1 μL RNase III at 37°C for 2 min to fragment the RNA molecules (Ion Total RNA-Seq Kit V2). The fragmented RNA was purified with 5 μL nucleic acid binding beads followed by the construction of whole transcriptome library including RNA adaptor ligation and reverse-transcription. The cDNA was purified, amplified, and checked by 2100 bioanalyzer (Agilent, USA). The library was sequenced using an Ion Torrent 318 chip on the Personal Genome Machine® (PGM, Life Technologies) according to the manufacturer's instructions. During analysis of sequence reads, the short (less than 30 bp) reads were filtered and low quality ($Q$ value<20) bases were trimmed. Remaining sequences were searched against the reference viral genome database (ftp://ftp- trace.ncbi.nlm.nih.gov/refseq/release/viral/) using BLAST+ 2.2.26 with an $E$-value cutoff of $10^{-3}$. The reads were *de novo* assembled and/or mapped to a reference DENV genome using Newbler 2.9 (Roche).

### 1.4 Complete genome sequencing

Total RNA was extracted using the Purelink RNA mini kit (Life Technologies) from C6/36 culture supernatant infected by isolated virus. Next, cDNA was produced using M-MLV reverse transcriptase (TaKaRa, Japan) with a specific reverse primer, and 13 primer pairs were used to generate overlapping amplicons spanning the entire genome. The PCR products were sequenced and assembled. The 5′ and 3′ untranslated regions (UTRs) of viral genome of the isolate were determined using a rapid amplification of either 5′ or 3′ cDNA ends (RACE) kit (Roche) following the manufacturer's recommendation. All primers are listed in Table S1 in Supporting Information.

### 1.5 Sequence alignment and phylogenetic analysis

Multiple sequence alignment was carried out employing the CLUSTAL W program [11,12]. Phylogenetic analyses based on the nucleotide sequence of complete envelope (E) gene of 37 DENV-2 were carried out by Neighbor-Joining method using MEGA version 5.05. The Neighbor-Joining trees were constructed by Tamura-Nei model with gamma-distribution of among-site. Sequences of the DENV-1 strain WestPac, DENV-3 strain H87 and DENV-4 strain H241 were used as outgroups.

## 2 Results

The patient was clinically diagnosed as severe dengue according to the 2014 guideline of National Health and Family Planning Commission of China and the 2009 guideline of WHO/TDR [4]. The laboratory parameters of the patient are shown in Table 1. Laboratory tests on peripheral blood examination showed severe thrombocytopenia occurred without increased hematocrit, and significant leukopenia, and hemoglobin decrease. The tourniquet test was positive. On abdominal ultrasonic examination, gall bladder thickening and multiple calculi were observed without hepatomegaly. The patient presented plasma leakage and severe organ involvement.

The acute phase serum was collected for nucleotide detection, and DENV-specific real-time RT-PCR showed positive results for DENV-2. However, DENV-specific IgM and IgG antibody were not detected in the patient serum (Table 1). Then, the acute phase serum was directly inoculated into C6/36 cells to isolate the pathogen. To rapidly identify virus genome sequence, high-throughput sequencing based on Ion Torrent was used to analyze the initial cell culture supernatants incubated with the patient sera. A total of 5240533 reads from raw sequencing data with average length of 106 bp were preprocessed by custom script. The home-made pathogen identification pipeline (AutoMetaAnalysis) detected 534 reads matched with DENV-2. The matched reads were then searched against NCBI nucleotide database using BLAST+ program and the results demonstrated that these sequences have the most similarity with DENV-2 isolate GZ40 (accession number JX470186). Using JX470186 as a reference, 2263 reads from the raw sequencing data were mapped to the reference genome and the average coverage was 23.45. The assembled virus genome sequence has an identity of 98% with the reference genome. This result indicates that DENV-2 was the causative agent of disease.

Five days post inoculation on the second passage in C6/36 cells, typical cytopathic effects (CPE) caused by DENV infection, characterized by cell fusion, enlargement, aggregation, swelling and vacuoles formation (Figure 1) were observed. The newly isolated DENV-2 was further identified by IFA, and the results showed that the patient's convalescent phase serum was fully reactive to the newly isolated virus (Figure 2). We named this clinical DENV-2 strain as GZ05/2014.

Further, routine RT-PCR was performed with virus culture to amplify the whole genome as previously described. The complete genome sequences of GZ05/2014 were finally obtained and submitted to GenBank under the accession number KP012546. The entire genome of DENV-2 strain GZ05/2014 is 10723 nt in length. The length of the 5′ and 3′-untranslated regions are 96 and 451 nt, respectively. The only open reading frame is predicted to encode a large polyprotein of 3392 amino acids.

Phylogenetic analysis based on E gene classified all DENV-2 isolates into five genotypes. The newly isolated DENV-2 strain GZ05/2014 belonged to the genotype Cosmopolitan, clustering closely with other DENV-2 isolates circulating in Guangdong, China from 2001 to 2013 (Figure 3). Other DENV-2 strains isolated in the 1980s in China belong to Asian I, Asian II, and American/Asian genotype, respectively. Especially, sequence alignment demonstrated that the E protein of GZ05/2014 has the 100% amino acid homology to strains GZ26199 (accession number KJ807-797), ZH413-2 (accession number KC131142) and GZ257 (accession number KJ277886) isolated from 2013, 2012, and 2001, respectively. The E protein of GZ05/2014 has above 99.8% amino acid homology to other strains isolated from the same region since 2001. This result demonstrates that the newly isolated DENV is not "complete novel" to Guangdong, the virus being there for a long time.

Finally, to identify whether there is any potential amino acid variation that would increase the virulence, the complete genome of GZ05/2014 was compared with other DENV-2 strains isolated in Guangdong, China. As shown in Table 2, all the critical amino acids retained identical among the

**Table 1** Laboratory parameters of the patient[a)]

| Parameter | Value | Reference value |
|---|---|---|
| Platelet count ($\times10^9$ $L^{-1}$) | 6 | 100–300 |
| WBC[1] ($\times10^9$ $L^{-1}$) | 2.5 | 4.0–10.0 |
| Hemoglobin (g $L^{-1}$) | 117 | 120–160 |
| Hematocrit (%) | 43 | 40–50 |
| Albumin (g $L^{-1}$) | 33 | 35–55 |
| LDH[2] (U $L^{-1}$) | 353 | 120–230 |
| ALT[3] (U $L^{-1}$) | 39 | 5–40 |
| AST[4] (U $L^{-1}$) | 78 | 5–40 |
| BUN[5] (mmol $L^{-1}$): | 5.9 | 2.9–80.2 |
| Creatinine (μmol $L^{-1}$) | 177 | 44–133 |
| Specific DENV-IgM | 2.36 | >11 |
| Specific DENV-IgG | 3.74 | >22 |
| Nucleic acid detection | DENV-2 | |

a) [1]WBC: white blood cells; [2]LDH: lactate dehydrogenase; [3]ALT: alanine transferase; [4]AST: aspartate transferase; [5]BUN: blood urea nitrogen.

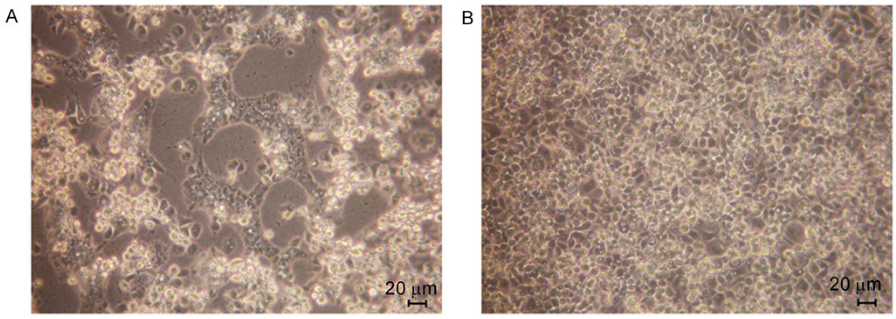

**Figure 1** The cytopathic effects (CPE) in C6/36 cells. A, C6/36 cells on 5 days post inoculation with the patient acute phase serum. B, Control C6/36 cells.

**Table 2** Critical amino acid mutations that determine viral replication and virulence of DENV[a)]

| Protein | Position | GZ05 (2014) | GZ26199 (2013) | ZH413-2 (2012) | GZ40 (2010) | GD01/03 (2003) | GD19 (2001) | GD08 (1998) | GD09 (1993) | D2-43 (1989) | D2-04 (1985) | Biological impacts | Reference |
|---|---|---|---|---|---|---|---|---|---|---|---|---|---|
| E | 67 | N | N | N | N | N | N | N | N | N | N | N67Q: decreased virus growth | [13] |
| | 71 | A | A | A | A | A | A | E | A | D | E | E71D: increased mouse neurovirulence | [14] |
| | 104 | G | G | G | G | G | G | G | G | G | G | G104S: decreased thermal stability | [15] |
| | 124 | N | N | N | N | N | N | N | N | N | N | N124D: reduced heparan sulfate binding affinity | [16] |
| | 126 | E | E | E | E | E | E | E | E | K | E | K126E: increased mouse neurovirulence | [14,17] |
| | 128 | K | K | K | K | K | K | K | K | K | K | K128E: reduced heparan sulfate binding affinity | [16] |
| | 135 | L | L | L | L | L | L | L | L | L | L | L135G: reduced replication in C6/36 cells | [18] |
| | 390 | S | S | S | S | S | S | N | S | N | N | N390H: increased mouse neurovirulence | [17] |
| | 398 | I | I | I | I | I | I | I | I | I | I | I398P: decreased the assembly and release | [19] |
| | 405 | T | T | T | T | T | T | T | T | T | T | T405P: decreased virus assembly and release | [19] |
| | 429 | F | F | F | F | F | F | F | F | F | F | F429P: decreased the assembly and release | [19] |
| | 436 | L | L | L | L | L | L | L | L | L | L | L436P: decreased the assembly and release | [19] |
| | 492 | M | M | M | M | M | M | M | M | M | M | M492V: increased replicative fitness in native mosquitoes | [20] |
| NS1 | 53 | G | NS* | G | G | G | G | G | G | G | G | G53D: temperature sensitivity | [21] |
| | 279 | F | NS* | F | F | F | F | F | F | F | F | L279F: increased replicative fitness in mosquitoes | [20] |
| NS4B | 52 | L | NS* | L | L | L | L | L | L | L | L | L52F: increased viral RNA synthesis | [22] |
| | 245 | N | NS* | N | N | N | N | N | N | N | N | N245S: increased replicative fitness in mosquitoes | [20] |
| NS3 | 250 | E | NS* | E | E | E | E | E | E | E | E | E250V: temperature sensitivity | [21] |
| NS5 | 200 | K | NS* | K | K | K | K | K | K | K | K | K200Q: increased replicative fitness in mosquitoes | [20] |
| | 290 | T | NS* | T | T | T | T | T | T | T | T | T290I: increased replicative fitness in mosquitoes | [20] |
| | 401 | R | NS* | R | R | R | R | R | R | R | R | R401K: increased replicative fitness in mosquitoes | [20] |

a) *, NS: not sequenced.

recent Guangdong DENV-2 isolates since 2001, which was in agreement with the phylogenetic analysis results. Especially, amino acid substitution at position 126 (K126E) in the E protein was seen in Guangdong isolates except D2-43, which was reported to have the neurovirulence phenotype in mice [23]. Amino acid substitutions at positions 71 and 390

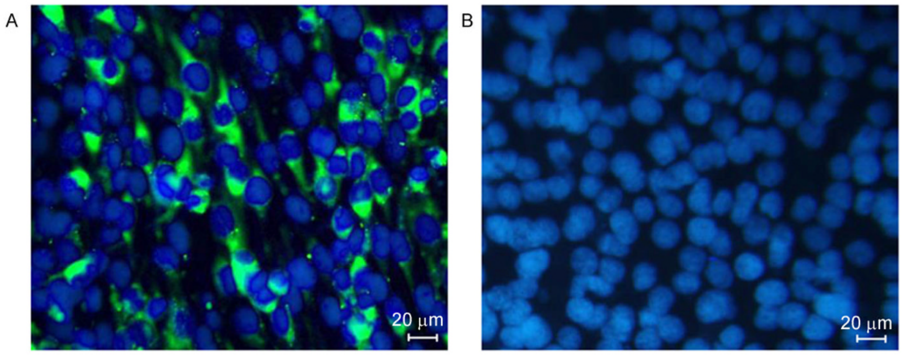

**Figure 2** Identification of the new DNEV-2 isolate by IFA. BHK-21 cells were infected with GZ05/2014 strain and 4 days after infection were fixed and incubated with the patient's convalescent phase serum. DAPI was used for nucleus staining. A, BHK-21 cells infected with GZ05/2014. B, Control BHK-21 cells.

in E protein were also identified; however, their biological effects remain unknown. A series of mutations increased DENV replication and viral fitness in mosquitoes have been well identified [20,24], while none of them was seen in GZ05/2014 and other Guangdong isolates (Table 2). Current sequence analysis failed to identify any amino acid mutations that would make an obvious connection between viral virulence and disease severity.

## 3 Discussion

In the present study, we isolated a DENV-2 clinical strain from a patient who was diagnosed as severe dengue during the large dengue fever outbreak in Guangdong, China. Rapid identification and characterization of the pathogen of an emerging epidemic is critical for disease control. Laboratory diagnosis of the case reported here was based on real-time RT-PCR, deep sequencing, and virus isolation. This multi-method analysis led to the reliable detection of DENV infection from the patient's serum. Virus culture is the gold standard for identification of DENV infections. Viral culture showed CPE indicative of virus growth, and the conventional real-time RT-PCR confirmed DENV-specific viral genome sequence was present. However, virus isolation in cell culture takes time and can be affected by multiple factors. Especially, DENV infection causes CPE slowly; some DENV strains even do not cause obvious CPE in cell culture. With the rapid development of HTS and high performance computing technologies, it is possible to identify the potential pathogen in a quick sequencing run without any pervious pathogen information, and the whole process including sample processing, library preparing, sequencing, and bioinformatics analyzing can be finished within 24 h. The HTS technology can be used as a fast and reliable method to detect viral pathogens in clinical samples, especially for the outbreak of endemic diseases. In this study, we for the first time identified the pathogen as DENV-2 from patient serum inoculation using HTS, and obtained 97% sequences of complete genome, without any prior information about the pathogen and without amplification using specific primers.

DENV-1 was predominant and responsible for the epidemics of dengue fever in Guangdong for decades [25]. However, the other three serotypes, DENV-2, DENV-3, and DENV-4, were all occasionally isolated in the past decade [26–30]. The co-circulation of multiple DENV serotypes increased the risk of secondary infection, and a severe dengue case due to secondary infection emerged in Guangdong province in 2011 [31]. Five DENV-2 genotypes have been described: Asian I, Asian II, American/Asian, Cosmopolitan and American [7]. According to polygenetic analysis results, the new isolate GZ05/2014 belonged to the Cosmopolitan genotype, clustering with other DENV-2 isolates circulating in China in recent 10 years (Figure 3). However, in early dengue epidemic before the 2000s, Asian I, Asian II and American/Asian genotypes have been observed, suggesting that a shift to Cosmopolitan genotype has occurred. Whether dengue is endemic in Guangdong remains to be demonstrated. Considering the fact that multiple genotypes of DENV-2 are co-circulating in South-Eastern Asian countries, the high homology and identical genotype of Guangdong DENV-2 strains suggest the possibility of establishment of local DENV-2 infection in Guangdong, China.

This unexpected large outbreak in China has raised the concern of the emergence of a "new" DENV with increased virulence or enhanced transmission. Most DENV virulence determinants have been well described, most of which locate at the E protein [13,15–19]. A mutation at 126 in the E protein hinge region from a negatively charged to a positively charged amino acid (K126E) has been implicated in the attenuation of viscerotropism for humans [14]. However, there were no reports about relationship between K126E substitution and dengue pathogenesis in humans. A few substitutions in NS1, NS4B, and NS5 protein were evidenced to increase viral replicative fitness in native mosquitoes, and contribute to DNEV genotype clade replacement [20,24]. None of these mutations existed in the new isolate GZ05/2014. In addition, the amino acid mutations affecting the viral temperature sensitivity and viral RNA synthesis [21,22] were also compared with the new isolate GZ05/2014, and no enhancement signal was observed. Most

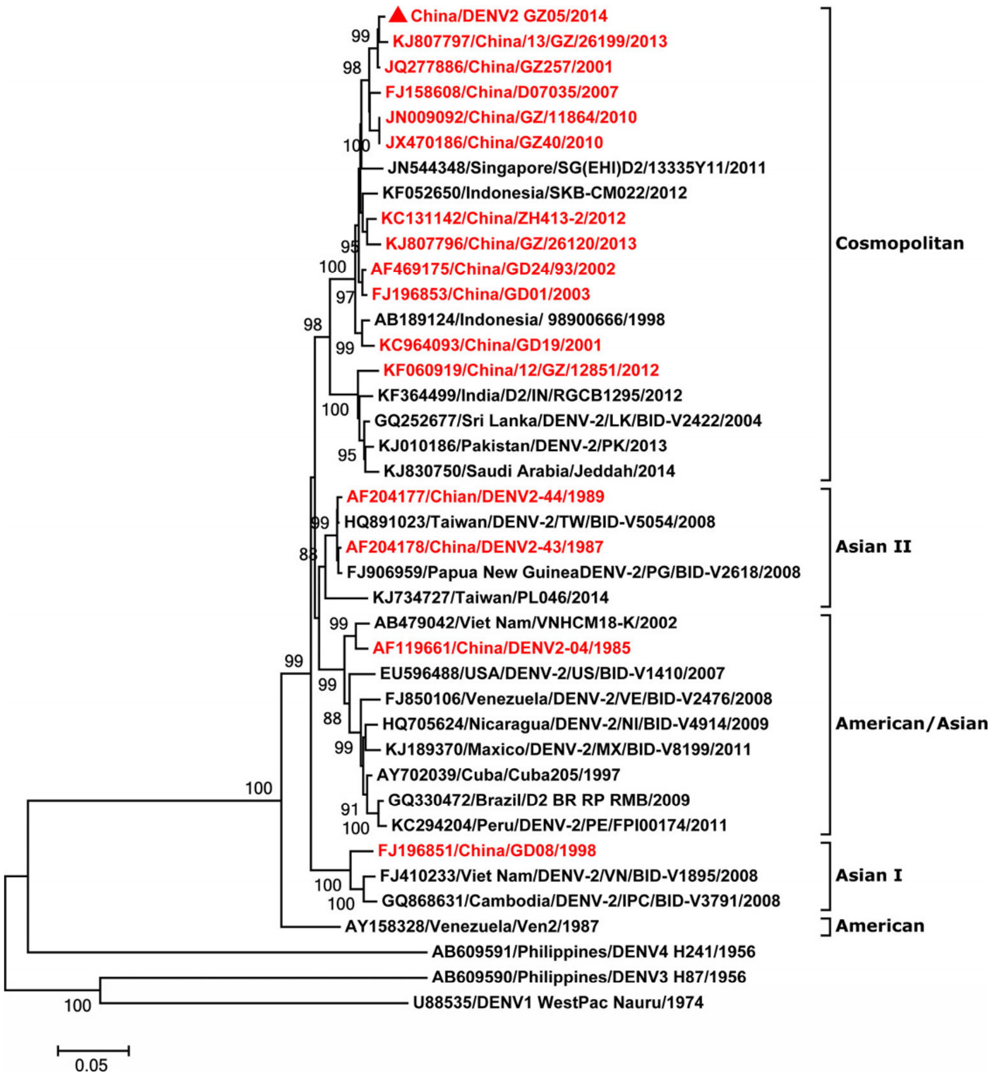

**Figure 3** Phylogenetic analyses of DNEV-2 isolates based on the complete envelope gene using the Neighbor-Joining method by Tajima-Nei model. GZ05/2014 isolated in this study is marked with red triangle, and all Chinese isolates are shown in red. DENV-1 strain WestPac, DENV-3 strain H87 and DENV-4 strain H241 were used as outgroups.

importantly, these critical amino acids are identical among all the DENV-2 isolates in Guangdong from 2001 to 2014, indicating that genetic characterization of DENV-2 strains circulating in Guangdong remains stable since its introduction in the 2000s. Current genomic analysis of GZ05/2014 has not yet explained the scale of this dengue epidemic and the severity of disease. Further phenotype characterization and virulence analyses using reverse genetic technology should be warranted in the future.

Currently, multiple serotypes of DNEV are circulating in Guangdong (unpublished data), and the origins and biological properties of these DENV isolates remain to be determined. Our study reports the first complete genome sequence of a clinical DENV-2 isolate during this severe outbreak in China. Extensive virological studies and comprehensive epidemiological investigation are underway to fight against DENV infection.

*We thank Mr. Nian QingGong and Mr. Xu YanPeng for technical assistance. This work was supported by Guangzhou Science and Technology Program for Public Wellbeing (2014Y2-00185), the Special Program of National Science and Technology of China (2013ZX10004-805), and the National Natural Science Foundation of China (81301491 and 31270974).*

1 Bhatt S, Gething PW, Brady OJ, Messina JP, Farlow AW, Moyes CL, Drake JM, Brownstein JS, Hoen AG, Sankoh O, Myers MF, George DB, Jaenisch T, Wint GR, Simmons CP, Scott TW, Farrar JJ, Hay SI. The global distribution and burden of dengue. Nature, 2013, 496: 504–507
2 Elling R, Henneke P, Hatz C, Hufnagel M. Dengue fever in children: where are we now? Pediatr Infect Dis J, 2013, 32: 1020–1022
3 Morens DM. Antibody-dependent enhancement of infection and the pathogenesis of viral disease. Clin Infect Dis, 1994, 19: 500–512
4 WHO/TDR. Dengue—Guidelines for diagnosis, treatment, prevention and control. 2009, http://www.who.int/tdr/publications/training-guideline-publications/dengue-diagnosis-treatment/en/index.html
5 Wu JY, Lun ZR, James AA, Chen XG. Dengue Fever in mainland China. Am J Tropical Med Hygiene, 2010, 83: 664–671
6 Li TG, Yang ZC, Luo L, Di B, Wang M. Dengue Fever epidemiological status and relationship with meteorological variables in Guangzhou, Southern China, 2007–2012. Biomed Environ Sci, 2013, 26: 994–997

7 Jiang L, Wu X, Wu Y, Bai Z, Jing Q, Luo L, Dong Z, Yang Z, Xu Y, Cao Y, Di B, Wang Y, Wang M. Molecular epidemiological and virological study of dengue virus infections in Guangzhou, China, during 2001–2010. Virol J, 2013, 10: 4

8 Wu LQ, Zhang XM, Che YC, Zhang Y, Tang SQ, Liao Y, Na RX, Xiong XL, Liu LD, Li QH. A cellular response protein induced during HSV-1 infection inhibits viral replication by interacting with ATF5. Sci China Life Sci, 2013, 56: 1124–1133

9 Yang EX, Zhao H, Zhang Y, Liu JS, Liao Y, Wang LC, Cui PF, Yang LX, Liu LD, Dong CH, Dong SZ, Shao CW, Jiang L, Sun L, Li QH. A comparative study of the characteristics of two Coxsackie A virus type 16 strains (genotype B). Sci China Life Sci, 2012, 55: 336–342

10 Deng YQ, Dai JX, Ji GH, Jiang T, Wang HJ, Yang HO, Tan WL, Liu R, Yu M, Ge BX, Zhu QY, Qin ED, Guo YJ, Qin CF. A broadly flavivirus cross-neutralizing monoclonal antibody that recognizes a novel epitope within the fusion loop of E protein. PLoS One, 2011, 6: e16059

11 Larkin MA, Blackshields G, Brown NP, Chenna R, McGettigan PA, McWilliam H, Valentin F, Wallace IM, Wilm A, Lopez R, Thompson JD, Gibson TJ, Higgins DG. Clustal W and Clustal X version 2.0. Bioinformatics, 2007, 23: 2947–2948

12 Geng H, Tan W. A novel human coronavirus: middle East respiratory syndrome human coronavirus. Sci China Life Sci, 2013, 56: 683–687

13 Bryant JE, Calvert AE, Mesesan K, Crabtree MB, Volpe KE, Silengo S, Kinney RM, Huang CY, Miller BR, Roehrig JT. Glycosylation of the dengue 2 virus E protein at N67 is critical for virus growth *in vitro* but not for growth in intrathoracically inoculated *Aedes aegypti* mosquitoes. Virology, 2007, 366: 415–423

14 Bray M, Men R, Tokimatsu I, Lai CJ. Genetic determinants responsible for acquisition of dengue type 2 virus mouse neurovirulence. J Virol, 1998, 72: 1647–1651

15 Butrapet S, Childers T, Moss KJ, Erb SM, Luy BE, Calvert AE, Blair CD, Roehrig JT, Huang CY. Amino acid changes within the E protein hinge region that affect dengue virus type 2 infectivity and fusion. Virology, 2011, 413: 118–127

16 Prestwood TR, Prigozhin DM, Sharar KL, Zellweger RM, Shresta S. A mouse-passaged dengue virus strain with reduced affinity for heparan sulfate causes severe disease in mice by establishing increased systemic viral loads. J Virol, 2008, 82: 8411–8421

17 Sanchez IJ, Ruiz BH. A single nucleotide change in the E protein gene of dengue virus 2 Mexican strain affects neurovirulence in mice. J Gen Virol, 1996, 77(Pt 10): 2541–2545

18 Huang CY, Butrapet S, Moss KJ, Childers T, Erb SM, Calvert AE, Silengo SJ, Kinney RM, Blair CD, Roehrig JT. The dengue virus type 2 envelope protein fusion peptide is essential for membrane fusion. Virology, 2010, 396: 305–315

19 Lin SR, Zou G, Hsieh SC, Qing M, Tsai WY, Shi PY, Wang WK. The helical domains of the stem region of dengue virus envelope protein are involved in both virus assembly and entry. J Virol, 2011, 85: 5159–5171

20 OhAinle M, Balmaseda A, Macalalad AR, Tellez Y, Zody MC, Saborio S, Nunez A, Lennon NJ, Birren BW, Gordon A, Henn MR, Harris E. Dynamics of dengue disease severity determined by the interplay between viral genetics and serotype-specific immunity. Sci Trans Med, 2011, 3: 114ra128

21 Butrapet S, Huang CY, Pierro DJ, Bhamarapravati N, Gubler DJ, Kinney RM. Attenuation markers of a candidate dengue type 2 vaccine virus, strain 16681 (PDK-53), are defined by mutations in the 5′ noncoding region and nonstructural proteins 1 and 3. J Virol, 2000, 74: 3011–3019

22 Grant D, Tan GK, Qing M, Ng JK, Yip A, Zou G, Xie X, Yuan Z, Schreiber MJ, Schul W, Shi PY, Alonso S. A single amino acid in nonstructural protein NS4B confers virulence to dengue virus in AG129 mice through enhancement of viral RNA synthesis. J Virol, 2011, 85: 7775–7787

23 Zhao W, Fan B, Hu Z, Chen S, Wang P, Yuan X, Li X, Yu M, Qin E, Yang P. Study on the determinants of suckling mice neurovirulence of dengue 2 virus. Sci China Ser C-Life Sci, 2003, 46: 95–103

24 Quiner CA, Parameswaran P, Ciota AT, Ehrbar DJ, Dodson BL, Schlesinger S, Kramer LD, Harris E. Increased replicative fitness of a dengue virus 2 clade in native mosquitoes: potential contribution to a clade replacement event in Nicaragua. J Virol, 2014, 88: 13125–13134

25 Wu W, Bai Z, Zhou H, Tu Z, Fang M, Tang B, Liu J, Liu L, Liu J, Chen W. Molecular epidemiology of dengue viruses in southern China from 1978 to 2006. Virol J, 2011, 8: 322

26 Zhang FC, Zhao H, Li LH, Jiang T, Hong WX, Wang J, Zhao LZ, Yang HQ, Ma DH, Bai CH, Shan XY, Deng YQ, Qin CF. Severe dengue outbreak in Yunnan, China, 2013. Int J Infect Dis, 2014, 27: 4–6

27 Zhao H, Yu XD, Zhang XY, Jiang T, Hong WX, Yu M, Hu FY, Zhu SY, Qin ED, Deng YQ, Qin CF, Zhang FC. Complete genome sequence of a dengue virus serotype 4 strain isolated in Guangdong, China. J Virol, 2012, 86: 7021–7022

28 Zhao H, Deng YQ, Hong WX, Yu XD, Jiang T, Yu M, Hu FY, Zhu SY, Li XF, Song KY, Qin ED, Zhang FC, Qin CF. Complete genome sequence of dengue virus serotype 2 Cosmopolitan genotype strain in Guangdong, China. J Virol, 2012, 86: 13808–13809

29 Jing QL, Yang ZC, Luo L, Xiao XC, Di B, He P, Fu CX, Wang M, Lu JH. Emergence of dengue virus 4 genotype II in Guangzhou, China, 2010: survey and molecular epidemiology of one community outbreak. BMC Infect Dis, 2012, 12: 87

30 Jiang T, Yu XD, Hong WX, Zhou WZ, Yu M, Deng YQ, Zhu SY, Qin ED, Wang J, Qin CF, Zhang FC. Co-circulation of two genotypes of dengue virus serotype 3 in Guangzhou, China, 2009. Virol J, 2012, 9: 125

31 Hong WX, Zhao H, Deng YQ, Jiang T, Yu XD, Song KY, Wang J, Tan XH, Yu M, Zhu SY, Qin ED, Tang XP, Zhang FC, Qin CF. Severe dengue due to secondary DENV-1 infection in mainland China. J Clin Virol, 2013, 57: 184–186

**Open Access** This article is distributed under the terms of the Creative Commons Attribution License which permits any use, distribution, and reproduction in any medium, provided the original author(s) and source are credited.

## Supporting Information

**Table S1** Primers used for DENV-2 sequencing reactions

The supporting information is available online at life.scichina.com and link.springer.com. The supporting materials are published as submitted, without typesetting or editing. The responsibility for scientific accuracy and content remains entirely with the authors.

## Electronic supplementary material

Supplementary material, approximately 206 KB.
