# Supplementary material for: Isolation and characterization of dengue virus serotype 2 from the large dengue outbreak in Guangdong, China in 2014
Source: Sci China Life Sci. 2014 Dec 11;57(12):1149–55. doi: 10.1007/s11427-014-4782-3 (PMC7089550; doi:10.1007/s11427-014-4782-3)
Supplement: Supplementary file 1 — Supplementary material, approximately 206 KB. [file 11427_2014_4782_MOESM1_ESM.pdf]

SCIENCE CHINA
Life Sciences

Supporting Information

December 2014 Vol.57 No.12: 1
doi: 10.1007/s11427-014-4782-3

Table S1 Primers used for DENV-2 sequencing reactions[a)]

Primer name	Sequence (5′–3′)
F1	agttgttagtctacgtggac
R1	ggcaggttgtttggcttc
F2	tggcatacaccataggaac
R2	tacatggagaaccgtccc
F3	cccatgcgaagaaacagg
R3	atgattcctttgatgtctcc
F4	aagttccaaccagaatccc
R4	tcgggtcctgagcatttc
F5	accaccgctaagatacagag
R5	ttgttattgacaggattggac
F6	aaccaacaagaaaaggagc
R6	ggggtttggtatcttattgg
F7	tccacccaggagcgggaaag
R7	ttccatcaaaacaccacc
F8	gaggagaagcaaggaaaac
R8	catccaatggcgagaagg
F9	ttacaacccagcaacccg
R9	tgttcgtcctgcttctacc
F10	gaagggaaagtagtggacc
R10	ggagattcctttccttgtc
F11	acacgagaacccaagaacc
R11	gcttgcgaacctgtcatc
F12	gagtcttcaaaagcattcagc
R12	ggggctcacaggtagcatag
F13[a)]	gaggaagaggcaggagtc
R13	agaacctgttgattcaacagc
5′RACE outer primer	gcaccaccaatctatgtcttc
5′RACE inner primer	ttcagcatccttccaatc

a) Primer F13 was also used as 3′RACE primer.

© The Author(s) 2014. This article is published with open access at link.springer.com
